# Preserving normal facial nerve function and improving hearing outcome in large vestibular schwannomas with a combined approach: planned subtotal resection followed by gamma knife radiosurgery

**DOI:** 10.1007/s00701-017-3194-0

**Published:** 2017-05-17

**Authors:** Roy Thomas Daniel, Constantin Tuleasca, Mercy George, Etienne Pralong, Luis Schiappacasse, Michele Zeverino, Raphael Maire, Marc Levivier

**Affiliations:** 1 Department of Clinical Neurosciences, Neurosurgery Service, and Gamma Knife Center, Lausanne University Hospital (CHUV), Rue du Bugnon 46, 1011 Lausanne, Switzerland; 2 Faculty of Biology and Medicine, University of Lausanne, Lausanne, Switzerland; 3 Medical Image Analysis Laboratory (MIAL), Lausanne University Hospital, Lausanne, Switzerland; 4 Signal Processing Laboratory (LTS 5), Ecole Polytechnique Fédérale de Lausanne, Lausanne, Switzerland; 5 Department of Otorhinolaryngology/Head and Neck Surgery, CHUV, University of Lausanne, Lausanne, Switzerland; 6 Radiation Oncology Service, Lausanne, Switzerland; 7 Institute of Radiation Physics, Lausanne University Hospital (CHUV), Lausanne, Switzerland

**Keywords:** Combined approach, Vestibular schwannoma, Surgery, Radiosurgery, Gamma knife

## Abstract

**Objective:**

To perform planned subtotal resection followed by gamma knife surgery (GKRS) in a series of patients with large vestibular schwannoma (VS), aiming at an optimal functional outcome for facial and cochlear nerves.

**Methods:**

Patient characteristics, surgical and dosimetric features, and outcome were collected prospectively at the time of treatment and during the follow-up.

**Results:**

A consecutive series of 32 patients was treated between July 2010 and June 2016. Mean follow-up after surgery was 29 months (median 24, range 4–78). Mean presurgical tumor volume was 12.5 cm^3^ (range 1.47–34.9). Postoperative status showed normal facial nerve function (House–Brackmann I) in all patients. In a subgroup of 17 patients with serviceable hearing before surgery and in which cochlear nerve preservation was attempted at surgery, 16 (94.1%) retained serviceable hearing. Among them, 13 had normal hearing (Gardner–Robertson class 1) before surgery, and 10 (76.9%) retained normal hearing after surgery. Mean duration between surgery and GKRS was 6.3 months (range 3.8–13.9). Mean tumor volume at GKRS was 3.5 cm^3^ (range 0.5–12.8), corresponding to mean residual volume of 29.4% (range 6–46.7) of the preoperative volume. Mean marginal dose was 12 Gy (range 11–12). Mean follow-up after GKRS was 24 months (range 3–60). Following GKRS, there were no new neurological deficits, with facial and hearing functions remaining identical to those after surgery in all patients. Three patients presented with continuous growth after GKRS, were considered failures, and benefited from the same combined approach a second time.

**Conclusion:**

Our data suggest that large VS management, with planned subtotal resection followed by GKRS, might yield an excellent clinical outcome, allowing the normal facial nerve and a high level of cochlear nerve functions to be retained. Our functional results with this approach in large VS are comparable with those obtained with GKRS alone in small- and medium-sized VS. Longer term follow-up is necessary to fully evaluate this approach, especially regarding tumor control.

## Introduction

The surgical management of benign skull-base tumors has evolved greatly in the last few decades, primarily in connection with the advancement of modern microsurgical techniques, which have significantly decreased the morbidity associated with the resection of these tumors [17, 18, 64]. On the other hand, several large series of radiosurgery for the treatment of small and medium benign skull-base lesions have demonstrated adequate long-term tumor control, along with very low neurological morbidity and improved preservation of functions, especially with regard to the risk for cranial nerve deficit, compared with surgery [20, 39, 41, 59, 66]. In large tumors, radical surgery alone yields a high risk for neurological deficit, and radiosurgery cannot be used safely as a first line of treatment because of the high risk for radiation-induced complications associated with large-volume tumors.

For vestibular schwannoma (VS), postoperative facial nerve and hearing dysfunction reported in the literature over several large series with surgery remains significant [1, 9, 31, 62]. Gamma knife surgery (GKRS) allows for optimal functional results in small and medium-sized VS, and has become a valuable alternative to upfront management in those cases [39, 41, 66]. Nowadays, patients with large VS have high expectations regarding the functional outcome of surgery, looking for results similar to those of patients treated with GKRS for smaller tumors.

Here, we present our experience with large VS using a treatment paradigm of a combined approach with microsurgery and GKRS, aiming to optimize the functional outcome for the facial and cochlear nerves.

## Materials and methods

### Study design and patient population

The study was designed as an open and prospective study. A case report form was generated prospectively for each patient at treatment time.

Between July 2010 and June 2016, a total of 257 patients with VS (all grades included) were treated at the Lausanne University Hospital, Switzerland. Whenever applicable, GKRS was used as the first-line treatment. Inclusion criteria for the combined approach were patients with Koos grade IV, who were not considered for upfront GKRS because of their size and/or significant brainstem compression. Furthermore, it is known that Koos grade IV tumors may vary significantly in tumor volume [42]. Therefore, the respective volumes are further detailed in Table 1 and in the Results section of the present paper (Demographic and preoperative data). Exclusion criteria for the present study were patients with VS treated with upfront GKRS during the same period of time, and patients with type II neurofibromatosis who benefitted from the combined approach.

**Table 1:** Demographic and surgical data and clinical assessment

Patient number	Sex	Age (years)	TV at surgery (cm^3^)	G-R class before surgery	G-R class after surgery	H–B grade before surgery	H–B grade after surgery
1	Male	61.2	16.1	5	5	I	I
2	Female	66.4	24	5	5	I	I
3	Female	34.4	11.3	1	1	I	I
4	Male	48.3	9.7	5	5	I	I
5	Male	44.2	10.4	3	3	I	I
6	Male	73.4	14.9	5	3	I	I
7	Male	69.9	27.4	5	5	I	I
8	Female	51.2	16.3	5	5	I	I
9	Male	34.4	34.9	5	5	I	I
10	Female	64.2	15.7	5	5	I	I
11	Female	32.5	25	1	5	I	I
12	Female	52.2	7.7	1	1	I	I
13	Female	48.3	19.9	1	1	I	I
14	Female	57.5	9.3	1	3	I	I
15	Male	73.8	10.1	5	3	I	I
16	Female	51.2	12.7	5	5	I	I
17	Female	46.2	5	3	3	I	I
18	Female	44.2	13.5	1	1	I	I
19	Female	34	13.5	5	5	I	I
20	Female	54.2	1.5	1	1	I	I
21	Male	66.7	7.9	4	4	I	I
22	Male	50	15.3	1	1	I	I
23	Female	51	7.4	3	2	I	I
24	Female	33	6	1	1	I	I
25	Male	55	15	5	5	I	I
26	Female	72	3.6	5	5	I	I
27	Female	41	3.2	1	1	I	I
28	Male	36	14.3	1	1	I	I
29	Female	40	3.6	3	2	I	I
30	Male	45	8.8	1	1	I	I
31	Female	39	5.2	1	3	I	I
32	Male	85	9.3	4	5	IV	I

*TV* tumor volume, *G–R* Gardner–Robertson, *H–B* House–Brackmann

In 32 patients (12.4%) with Koos grade IV who met the inclusion criteria planned subtotal microsurgical resection was performed within the framework of a combined approach. This represents the total number of patients receiving open surgery for VS of any size during the study period. Among the 32 cases, 3 patients treated primarily with GKRS continued to progress and were considered as GKRS failures after 3 years of follow-up; they were operated on using the same combined approach, and were therefore included in this study. Likewise, 1 patient previously treated elsewhere with fractionated radiation therapy, and 1 patient treated with LINAC radiosurgery, were also considered as failures, were managed using the same combined approach, and were included in this study. All the surgical resections were performed by the first author (RTD), and all GKRS were performed by the last author (ML).

Follow-up included patients and tumor characteristics analyzed at baseline (before surgery) and at regular follow-up intervals both after surgery and after GKRS; clinical assessment and magnetic resonance imaging (MRI) were performed after surgery and at 6, 12, 24, and 36 months after GKRS (unless otherwise clinically indicated). Parameters included age, symptoms at initial presentation, and the neurological examination. The facial nerve function was classified using the House–Brackmann (H–B) grading scale [26]. During the follow-up period, it was always performed independently by the second (CT, neurosurgeon) and third authors (MG, ENT), who were not involved in the surgery.

The hearing assessment included pure tone audiometry (PTA) and speech discrimination score (SDS) and was graded according to the Gardner–Robertson (G–R) classification scale [16].

Maximal diameter was defined as the largest diameter after measuring all three axes (lateral, antero-posterior and supero-inferior). The volume was defined by automatic segmentation using Leksell GammaPlan® planning software (Elekta Instruments AB, Stockholm, Sweden) on all MR data, including the preoperative images. The tumor size (maximal diameter) and volume were measured on the serial MRI [35].

### Surgical technique

All patients were operated on using the retrosigmoid approach in the lateral decubitus position, as previously described by several authors [5, 7, 8, 11, 62]. Intraoperative neuromonitoring (IOM) is an essential part of this surgery. The two main goals of IOM are to precisely define the anatomical location of the nerves in the cerebellopontine angle and to preserve the functional integrity of the facial and cochlear nerves. Bipolar electrodes are placed in the frontalis, orbicularis occuli, orbicularis oris, and mentalis muscles. Glossopharyngeal and vagus nerves are monitored using contact electrodes incorporated in the endotracheal tube (Xomed®; Medtronic, Jacksonville, FL, USA). Electrodes inserted into the trapezius muscle are used for monitoring the eleventh nerve function using an NIM-Eclipse® (Medtronic) intraoperative monitoring system with free-running electromyography (EMG) and compound muscle action potential (CMAP) after direct nerve stimulation using monopolar 1-Hz, monophasic negative 200-μs duration electrical stimulation. Alert criteria are a stimulation response at low stimulation intensity indicating nerve proximity or spontaneous EMG bursts lasting more than 30 s on the free-running EMG. In patients with preserved hearing, brainstem auditory evoked responses (BAER) are used to monitor the integrity of the cochlear nerve.

**Fig. 1:**
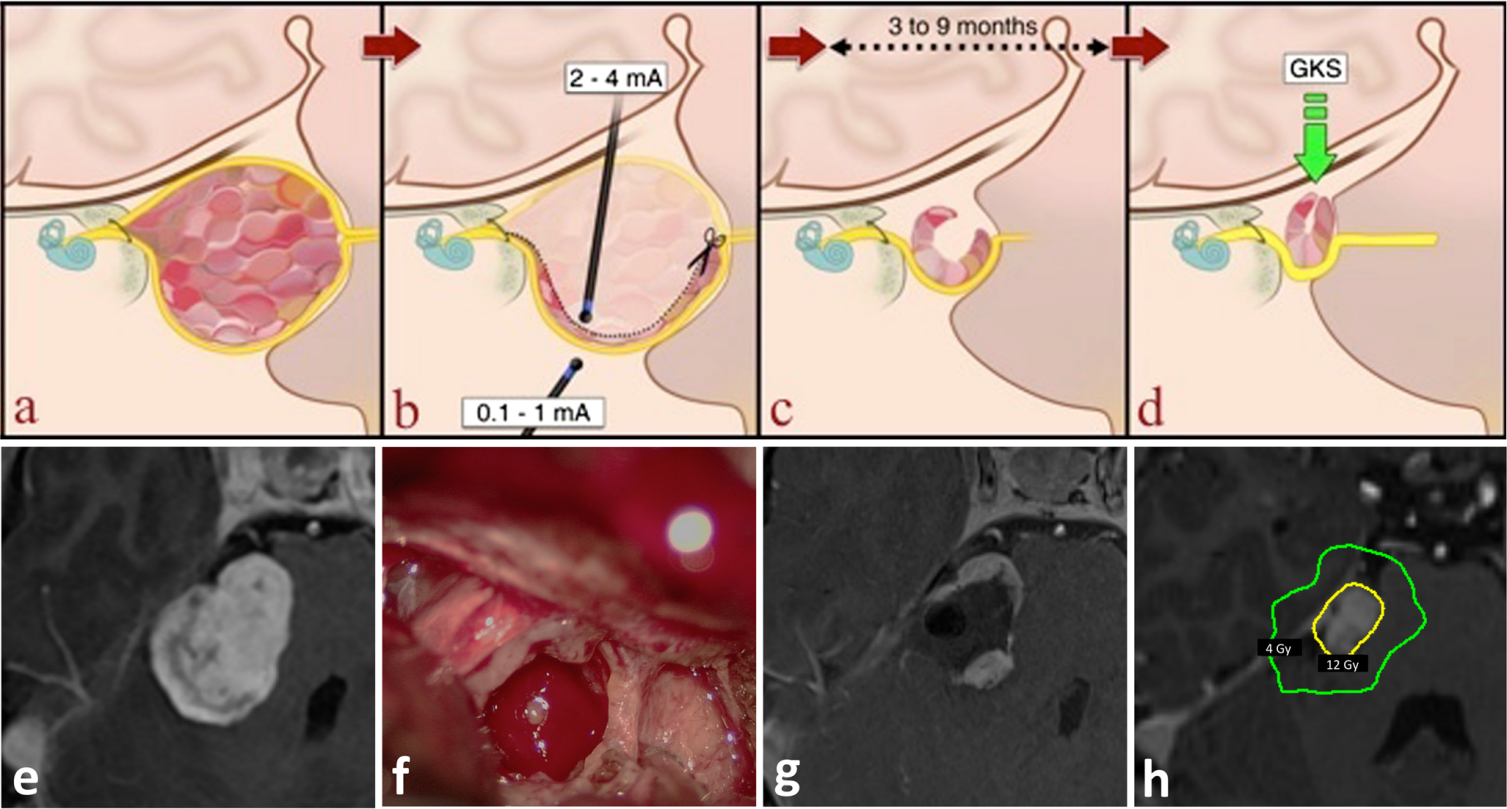
Schematic representation of a planned subtotal resection and gamma knife surgery (GKRS) planning of large vestibular schwannoma (VS).* Upper part*: illustrations of** a** the tumor in the coronal plane surrounded by the nerves that enter the internal auditory canal, and its relationship to the brainstem,** b** the internal decompression of the tumor guided by stimulation of the nerve, both from the external side of the capsule and through the residual capsule,** c** the progressive closure of the residual capsule and relief of brainstem compression,** d** the final size and shape of the residual tumor for GKRS.* Lower part*:** e** preoperative MRI,** f** intraoperative view,** g** immediate postoperative MRI (24 h after surgery), and** h** MRI at the time of GKRS with the dosimetry (prescription isodose volume colored in* yellow*, and the 4-Gy isodose line in* green*, while the cochlea is colored in* magenta*)

The durotomy is performed in a linear fashion 5–8 mm from the border of the sigmoid sinus and the transverse sigmoid junction. The lateral cerebellomedullary cistern is opened to let out the CSF and relax the cerebellum. The tumor is then visualized in the cerebellopontine angle. The posterior capsule of the tumor is stimulated to look for an aberrant facial nerve course, after which the posterior capsule is opened. Tumor decompression is performed with an ultrasonic aspirator and the tumor capsule progressively mobilized from the cerebellum and brainstem. The microsurgical strategy for a planned subtotal resection differs from that for a maximized or total excision in several aspects. When a total excision is planned for, the plane between the tumor and the facial nerve is identified by visual and electrophysiological means. This plane is then followed from the root entry zone to the internal auditory meatus (IAM) to remove the capsule (after adequate internal decompression) in its entirety. The arachnoidal relationships of the facial and cochlear nerves with the tumor capsule is a matter of debate and possibly depends on the whether the tumor is of an epi- or subarachnoid origin. In the author’s earlier experience with total excisions of large VS, it was very difficult, if not impossible, to identify arachnoid between the tumor capsule and the facial and cochlear nerves and all these structures are contained within a single cistern. The damage to these nerves is very likely to be due to a combination of the manipulation of extremely thinned out nerves and compromise of the vascular supply to these nerves. The present strategy (in this series) of a planned subtotal tumor excision takes advantage of the fact that this plane is not entered into and thus obviating these factors that lead to neural compromise. The thinned out capsule of the tumor is progressively excised till the superior and inferior border of the facial nerves are identified by IOM. This is performed by stimulation of the external part of the capsule with a current smaller than 1 mA. This current is progressively decreased till the smallest current that evokes a positive response is identified. This parameter is then used as a standard during the entire procedure, whenever the external capsule is stimulated. The tumor capsule covering the facial nerve is stimulated from within the tumor at currents of 4–5 mA and progressively the current is decreased till 2 mA. In our experience, if a positive stimulation is achieved internally at 2 mA, the thickness of the residual capsule is around 2–3 mm. The capsule is also turned and inspected (and stimulated) from its external surface to estimate the thickness (Fig. 1a, b). The free-running EMG responses are used during surgery for alert criteria as described above. Combining electrophysiological responses with visual inspection gives the best chance of achieving a uniformly “small” thickness residual capsule that protects the facial nerve course from surgical damage. At the end of surgery, facial nerve integrity is tested using direct brainstem stimulation at the level of the facial root entry zone, and in our series, facial nerve response could be elicited by stimulation less than 1 mA in all patients. In addition, there was no persistent EMG-bursting activity at the end of surgery, indicating the absence of an axonal lesion of the facial nerve. The opening of the internal auditory meatus (IAM) is not part of our strategy within the framework of this combined approach; the IAM is not opened because this is the region where the nerves are most vulnerable. The only exception was for one case, where the patient presented with a progressive facial nerve paresis preoperatively, due to a large multilobulated tumor component within the meatus. The decompression of the tumor within the meatus allowed for complete facial nerve recovery (H–B grade IV improved to grade I).

Cochlear nerve preservation is more difficult to achieve, as there are no stimulation methods that allow clear electrophysiological identification of the cochlear nerve for large tumors. BAER is used in a continuous manner with defined alert criteria such as reduction of peak III amplitude of more than 50%. The identification of the facial nerve course from the internal auditory meatus to the root entry zone at the brainstem allows for prediction of the course of the cochlear nerve, which is presumably located caudally (inferiorly) to the facial nerve. The thickness of the residual capsule over the cochlear nerve is kept to a similar degree as that achieved with the facial nerve, guided by a visual assessment.

Early postoperative MRI is performed as a baseline for evaluating the volume and shape of the residual tumor, and GKRS is planned a few months later, after a new MRI has been performed, expecting that the residual capsule has closed on itself gradually, owing to brain pulsations and reduction of the mass effect (Fig. 1c, d).

### Gamma knife radiosurgery technique

The Leksell G stereotactic frame (Elekta instruments AB, Stockholm, Sweden) was fixed under local anesthesia and mild sedation, with specific attention given to avoiding the area of the previous posterior fossa craniotomy. All patients underwent stereotactic MRI (1.5 Tesla, Siemens, Erlangen, Germany) and computed tomography (CT) with bone windows. The MR sequences used are T1-weighted with and without gadolinium contrast medium and T2 CISS, without contrast medium (in small remnants) and/or with contrast medium (in larger remnants); T2 CISS with contrast medium helps to differentiate between the nerves and the tumor [24]. Target definition and treatment dosimetry were performed using Leksell GammaPlan® planning software version 10 or 11. The modiolus of the cochlea was defined on bone CT images and the dose received was further calculated (as the dose received by the first 1% of the volume, and as the maximum dose). If this dose was more than 4 Gy in patients with useful hearing, additional sector blocking was used to ensure that optimal dosimetry results are obtained, as we routinely perform in patients undergoing upfront GKRS for smaller tumors [41, 60]. The stereotactic irradiation was delivered using the Leksell Gamma Knife® Perfexion™ (Elekta Instruments AB, Stockholm, Sweden). The procedure was performed on an ambulatory basis.

### Data analysis

All collected data were analyzed for the whole group (32 patients). Moreover, data were analyzed separately in the subgroup of patients with serviceable hearing before surgery and in which preservation of cochlear nerve function was attempted (group A, G–R 1–3, *n* = 17), and in patients with no residual preoperative hearing (group B, G–R 4 and 5, *n* = 15).

### Statistical analysis

All statistical analyses were conducted using Stata (STATA version 11 (STATA, College Station, TX, USA) and GraphPad Prism software 5.02. For tumor control after GKRS, survival over time was examined using the Kaplan–Meier method. Patients censoring occurred at the moment of failure (for the 3 cases who needed a second combined approach), or at last follow-up otherwise (for stability or decrease in size during follow-up course respectively). For the two-sample* t* test (continuous variables), a *p* value of <0.05 was considered statistically significant. For categorical variables, Chi-squared test was performed. Owing to insufficient sample size no multivariate analysis was carried out.

## Results

### Demographic and preoperative data

Thirty-two patients (13 male and 19 female) with large VS have undergone planned partial microsurgical resection with this approach, followed by GKRS. The mean age at the time of surgery was 51.7 years (range 32.5–85).

The clinical presentation was progressive hearing loss (20 cases, 62.5%), sudden hearing loss (2 cases, 6.25%), gait problems (2 patients, 6.25%), trigeminal nerve symptoms (2 cases, 6.25%), tinnitus (1 case, 3.12%), vertigo (4 cases, 12.5%), and was an incidental finding in 1 case (3.12%). The VS was solid in 29 cases (90.6%), and mixed (solid and cystic) in 3 patients (9.7%).

All patients had normal facial nerve function (H–B I) before surgery, except for one patient who was had H–B IV before surgery. All patients underwent preoperative hearing evaluation consisting of tonal and vocal audiometry. Hearing status according to the G–R scale is displayed in the Table 1.

The mean presurgical tumor volume was 12.5 cm^3^ (range 1.47–34.9) and the mean presurgical maximal diameter was 33.2 mm (median 35, range 20–45). The mean tumor volume was 9.8 cm^3^ (range 1.47–25) in group A, and 15.4 cm^3^ (range 3.6–34.9) in group B (*p* = 0.03, two sample* t* test). The mean maximal diameters in groups A and B were 30.6 (median 30, range 20–42) and 36.1 (median 37.7, range 26–45) respectively (*p* = 0.01, two sample* t* test).

### Postoperative data and functional outcome

The mean follow-up after surgery was 29 months (median 24, range 4–78). One patient was lost to follow-up owing to relocation to another country.

Postoperative clinical status showed normal facial nerve function (H–B I) in all operated patients (i.e., 100% preservation of normal facial nerve function, including the recovery of the facial function of the patient who presented with H–B IV preoperatively).

Cochlear nerve preservation surgery was attempted for the 17 patients in group A who presented with G–R class 1 (13 patients) or G–R 3 (4 patients) before surgery. Of the 13 patients in G–R class 1, postoperative audiogram showed that 10 (76.9%) remained in G–R class 1, two were in G–R class 3 (15.4%), and one (7.7%) lost hearing (G–R class 5). Of the 4 patients in G–R class 3, postoperative audiogram showed that 2 remained in the same class after surgery, and that PTA improved in 2 of them during follow-up (G–R class 2). In summary, postoperative hearing status in group A was identical or better in 14 out of 17 patients (82.3%) and useful postoperative hearing (G–R 1) in this group was maintained in 10 out of 13 patients (76.9%).

In group B, i.e., the 15 patients with no serviceable hearing before surgery (G–R 4 and G–R 5), of the 13 patients presenting with G–R 5, two improved to G–R 3 after surgery; from the two patients in G–R 4 preoperatively, one remained in the same hearing class and one passed to G–R 5 after surgery.

The mean PTA values for patients with serviceable hearing before surgery were 37.2 dB (median 40, range 5–80) and after surgery they were 46.4 (median 45, range 5–120; *p* = 0.3, two sample* t* test). At 6 months after GKRS, the mean value was 49.6 (median 45, range 105–120; *p* = 0.55, two sample* t* test, compared with the values immediately after surgery). At 12 months after GKRS the mean values were 61.7 (median 66.2, range 15–120; *p* = 0.35, two sample* t* test, compared with 6 months after GKRS) and at 24 months they were 62.7 (median 70, range 8.7–120; *p* = 0.96, two sample* t* test, compared with 12 months after GKRS; *p* = 0.42, two-sample* t* test, compared with baseline, before GKRS). Of note, as the follow-up period is still short in some cases, the number of observations before surgery, after surgery, and at 6 months after GKRS was 15 and dropped to 8 at 1 year, to 5 at 2 years, and to 2 at 3 years after GKRS.

One of the 2 patients, who presented with secondary trigeminal neuralgia before surgery, had transient facial hypoesthesia following surgery. Trigeminal neuralgia disappeared in both cases. One patient had a transient vagus nerve deficit, with full recovery after 6 months. No other neurological deficits were encountered after surgery.

### Gamma knife radiosurgery data and outcome

**Fig. 2:**
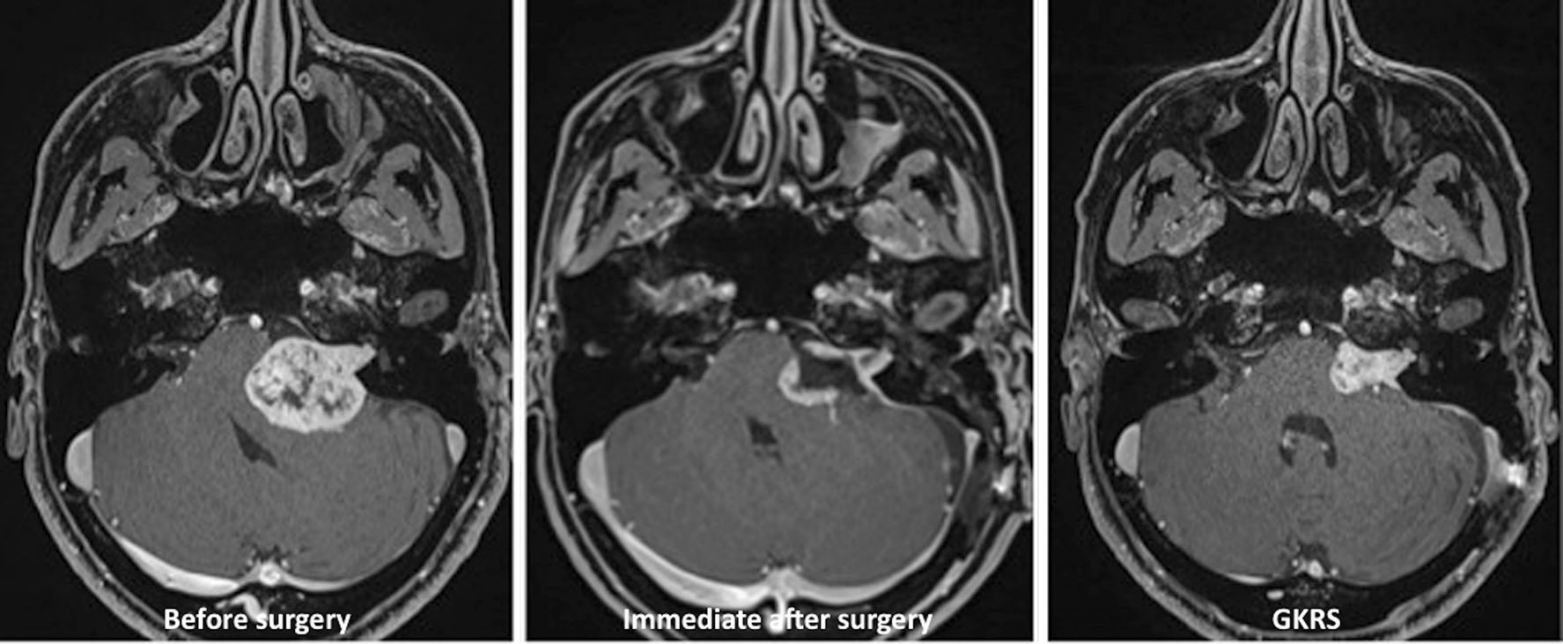
Representative axial images of T1-weighted contrast-enhanced MRI showing the preoperative size of the tumor (*left*), the residual capsule immediately after surgery (*center*), and the folding of the capsule and reduction of mass effect on the brainstem at the time of GKRS (*right*), which results in a compact and small target volume

Although GKRS was generally planned to take place at around 6 months after surgery, the exact timing depended on the suitability of the target for GKRS and on the shape of the residual tumor capsule on follow-up MRI. One expects that the open residual capsule at the end of surgery gradually closes on itself owing to brain pulsations and reduction of the mass effect; ideally, a complete closure, therefore, converts a large debulked VS into a small, more globular tumor with a shape and size that become more suitable for GKRS (Fig. 2). Most patients had early (within 48 h) postoperative MRI, and all patients underwent MRI 3–4 months after surgery. Depending on the size and shape of the residual tumor at that time, GKRS was scheduled, or patients were further followed with MRI. Three patients needed staged surgery (i.e., they were operated on twice) before GKRS, because the residual volume after the first surgery was still considered too large for safe GKRS.

All 32 patients have undergone GKRS after surgery. The mean duration between surgery and GKRS was 6.3 months (median 6, range 3.8–13.9 months). The mean tumor volume at the time of GKRS was 3.5 cm^3^ (range 0.5–12.8); this corresponded to a mean residual volume of 29.4% (range 6–46.7) of the preoperative volume (Table 2).

**Table 2:** Gamma knife radiosurgery dosimetric data

Patient number	TV at GKS (cm^3^)	Percentage of residual tumor	PIV at GKS (cm^3^)	Prescribed dose (Gy; at the 50% isodose line)
1	0.963	6	1.38	12
2	2.4	10	3.01	12
3	4	35.4	4.75	12
4	3.2	33	3.48	11
5	3.3	31.7	3.83	11
6	0.537	3.6	0.792	12
7	7.6	27.7	8.87	12
8	6.6	40.5	7.56	12
9	12.8	36.7	13.41	12
10	7.05	44.9	7.59	12
11	7.7	30.8	7.8	12
12	3.6	46.7	4.15	12
13	4	20.3	4.54	12
14	3.38	36.3	3.69	12
15	1.62	16	1.94	12
16	4.04	31.8	4.5	12
17	1.38	27.6	1.68	12
18	2.5	18.5	3.34	12
19	2.42	17.9	2.92	12
20	0.519	35.3	0.665	12
21	1.44	18.3	1.91	12
22	3.62	23.6	4.23	12
23	2.17	29.4	2.99	12
24	1.24	20.8	1.6	12
25	4.74	31.6	5.6	12
26	1.29	35.4	1.64	12
27	1.5	46.6	1.58	12
28	5.51	41.9	7.01	12
29	0.721	20.3	0.981	12
30	5.57	63.2	6.54	12
31	2.12	40	2.57	12
32	1.9	20.5	2.482	12

*PIV* prescription isodose volume

**Fig. 3:**
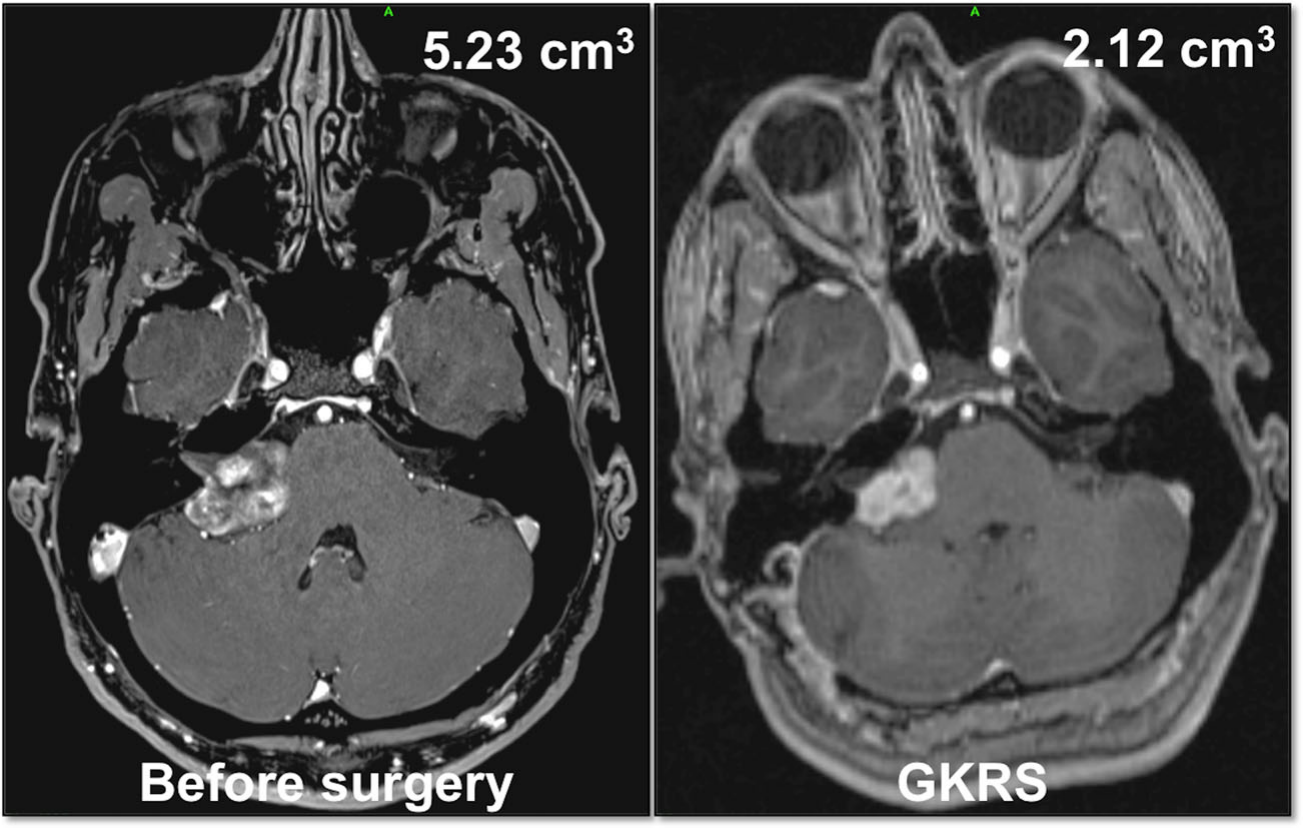
Illustrative case in group A (cochlear nerve preservation attempt at surgery). A 39-year-old female patient presented with minimal loss of hearing (Gardner–Robertson class 1) before surgery. The T1-weighted axial contrast-enhanced MRI showed a large VS, with a preoperative tumor volume of 5.23 cm^3^ (*left*). The tumor volume was reduced to 2.12 cm^3^ (i.e., 40% of the preoperative volume) at the time of GKRS. The patient remained in Gardner–Robertson 1 after surgery and had normal facial nerve function (House–Brackmann I)

**Fig. 4:**
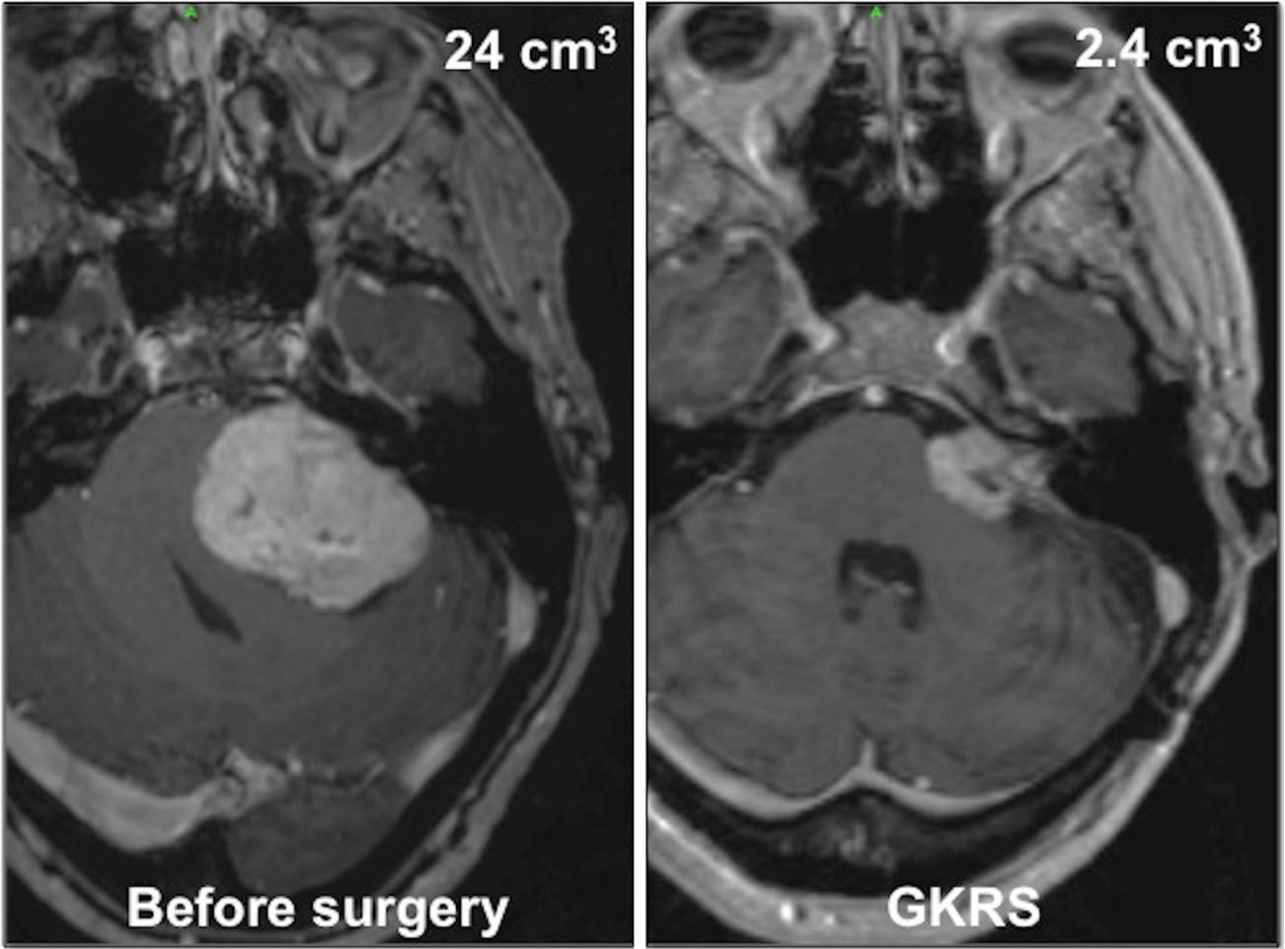
Illustrative case in group B (no attempt at cochlear nerve preservation at surgery). A 67-year-old woman presented with loss of hearing (Gardner–Robertson class 5) and gait problems. The T1-weighted gadolinium-enhanced MR axial images showed very large VS with significant brainstem compression, with a preoperative tumor volume of 24 cm^3^. The tumor volume was reduced to 2.4 cm^3^ (i.e., 10% of the preoperative volume) at the time of GKRS. The facial nerve function remained normal (House–Brackmann I)

The volume at the time of GKRS, when compared with the presurgical volume, was bigger when cochlear nerve preservation was attempted (group A, 33.4% of pre-surgical volume; group B, 25.1% of pre-surgical volume), as exemplified in Figs. 3 and 4. Although these results did not reach statistical significance, despite showing a strong tendency (*p* = 0.059, two sample* t* test), there was a trend toward the tumors in Group A being smaller and the tumor remnant after GKRS to be larger in this group. This is essentially related to the fact that a larger tumor capsule needs to be left behind to preserve cochlear nerve function.

The mean prescription isodose volume was 4 cm^3^ (range 0.6–13.4). The mean number of isocenters was 20 (median 20, range 7–33) and the mean marginal prescription dose was 12 Gy (median 12, range 11–12 Gy); there was no dose reduction for patients with previous GKRS or other forms of radiation.

The mean conformity index for the combined series was 0.98 (range 0.92–1.00), the mean selectivity index was 0.81 (range 0.66–0.99), and the mean gradient index was 2.7 (range 2.4–3.0).

Out of the 32 treated patients, 20 had at least 1 year of follow-up post-GKRS. The global mean follow-up after GKRS was 24 months (median 24 months, range 3–60). Following GKRS, no new neurological deficits were encountered and hearing remained stable in all patients with preserved hearing post-surgery.

### Tumor control after combined approach

Three patients (9.4%) had continuous growth of the VS after the combined approach, with clinical worsening (see below), and have been considered as failures, at 2.6, 2, and 1.25 years after GKRS respectively. Thus, the actuarial local tumor control rate (LTC) at 1 year is 94%, at 2 years 88%, and at 2.6 years became 77% and further remained stable over time.

**Fig. 5:**
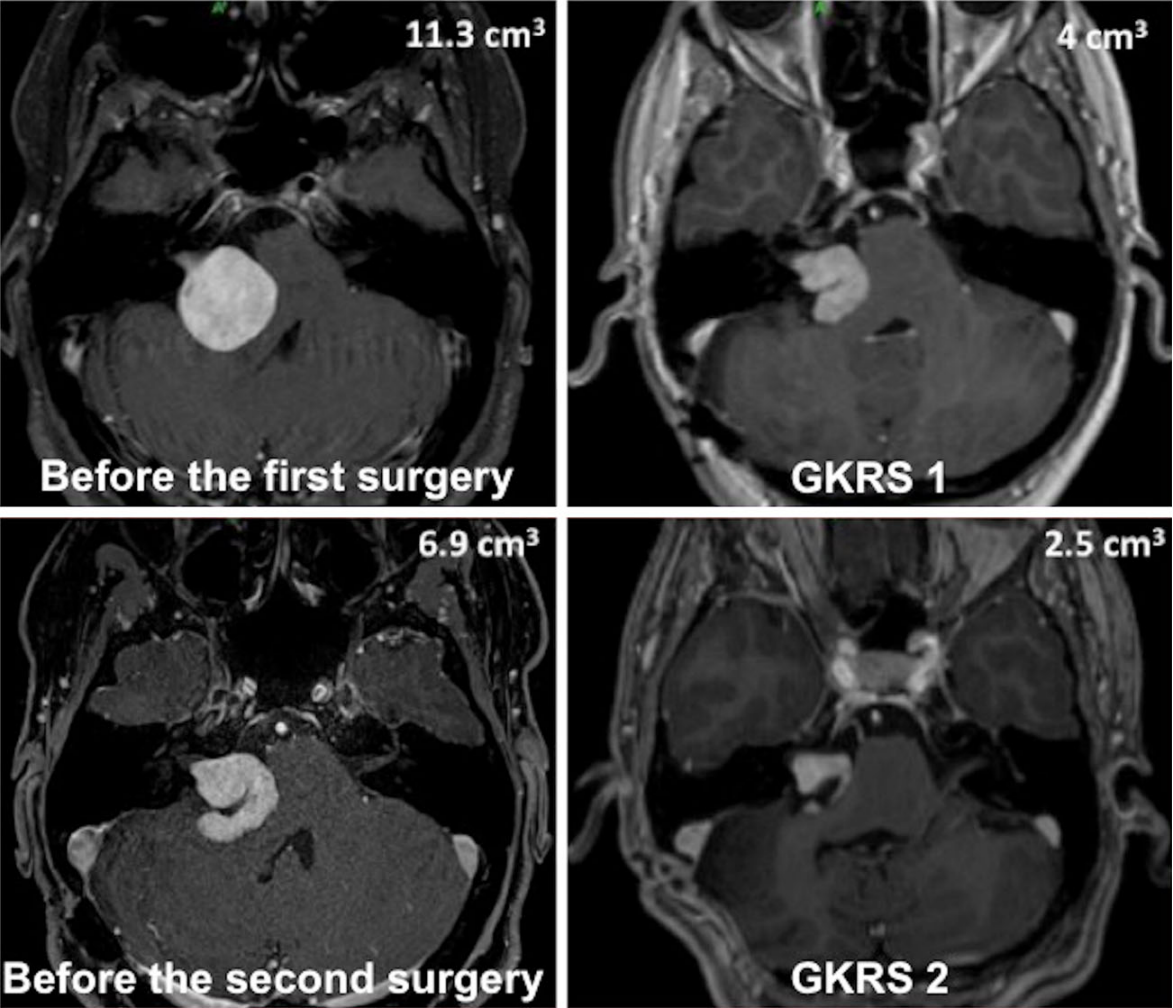
Illustrative case of a failure after a combined approach, which was re-treated using the same approach. A 37-year-old woman presented with minimal hearing loss (Gardner–Robertson class 1) and secondary intractable trigeminal neuralgia. T1-weighted gadolinium-enhanced MR axial images showed a large VS with significant brainstem compression that had a preoperative tumor volume of 11.3 cm^3^ (*upper image left*); the tumor volume was reduced to 4 cm^3^ following the surgery and at the time of the first GKRS (*upper image right*); the trigeminal neuralgia disappeared and the patient retained functional hearing (Gardner–Robertson class 1). The tumor continued to grow and at 2 years after GKRS, the patient developed recurrent trigeminal neuralgia and gait instability; the MRI showed that the tumor volume had continued to increase to 6.9 cm^3^ (*lower left*) and a second surgical resection was decided upon. The tumor volume was reduced to 2.5 cm^3^ following the second surgery and at the time of the second GKRS (*lower right*). She remains asymptomatic, with normal facial nerve (House–Brackmann I) and hearing function (Gardner–Robertson 1) following the two surgeries and the two GKRS procedures

The three cases that were considered to be failures have undergone a new combined approach. The first case (patient #3 in the series, Fig. 5) was considered a failure 2 years after the first GKRS. She presented with intractable, recurrent secondary trigeminal neuralgia and a new MRI showed a continuous increase in tumor volume. She has been further re-operated 2 years after the first GKRS. She retained hearing (G–R class 1) at 2 years after the second GKRS, and facial nerve function remained normal (H–B I). The second case (patient #9 in the series) was considered a failure 2.6 years after GKRS. He presented with recurrent gait instability, and the new MRI displayed a significant increase in tumor volume. He has been further reoperated 2.6 years after the first GKRS. He already had no residual hearing before the first combined approach. Facial nerve function was normal (H–B I) after the 2nd surgery. The third case (patient #11 in the series) was considered a failure 1.25 years after GKRS. She presented with intractable headaches and serial MRI controls that displayed a continuous major increase in tumor volume. She was further reoperated 1.25 years after GKRS. She had already lost hearing after the first combined approach, and facial nerve function remained normal (H–B I) after the new surgery.

The ages of these patients were 34.4, 34.4, and 35.2 years (mean 34.6) respectively, i.e., among the youngest patients in our series. The volumes at the time of GKRS were 4, 7.7, and 6.6 cm^3^ respectively. Review of the pathological condition did not show any change in the histopathological grade between the two surgeries, and there were no signs of malignant transformation.

The distinction between TTE and tumor growth is not always easy. In our cases, the clinical condition and symptoms, in addition to the continuous growth on MRI, were in favor of treatment failure, and we decided to perform a new combined approach in these patients. As illustrated in the literature, the decision whether to continue to wait-and-scan or to act by a new therapeutic means is always a clinical decision, and “never just a matter of volume measurements” [43, 47, 58].

## Discussion

The optimal management of VS remains a matter of debate. For small to medium-sized lesions, the options are radiosurgery, microsurgery or a “wait and scan” approach. Small to medium-sized (Koos grade I to III) symptomatic lesions can be treated either by surgery or by radiosurgery based on surgeon preference or availability of expertise. For small to medium-sized tumors, comparative studies suggest that GKRS might compare favorably with microsurgery, with a high degree of local tumor control, a much lower rate of facial nerve palsy, and a much higher rate of serviceable hearing preservation [44, 46, 51, 52, 55]. Recent clinical trials have suggested pro-active radiosurgical treatment, even in small Koos grade I intracanalicular tumors [57].

Large symptomatic VS are generally considered to be an indication for microsurgical excision, except in cases with significant co-morbidities contraindicating open surgery. The management remains challenging, especially with regard to facial and cochlear nerve outcomes. The expectations of the patients with large VS and their referral doctors have increased, especially in the current context of high-quality outcomes that are available for patients with smaller lesions treated with GKRS. Thus, patients with large VS are often referred to centers equipped with radiosurgery, expecting results comparable with those reported in patients with smaller lesions. The change in our treatment paradigm for the treatment of large VS was made specifically to address those issues.

In our series, only 32 out of 257 patients (12.4%) had Koos grade IV tumors, which may seem a comparably low percentage compared with other studies. However, if we look at recent studies with approaches comparable with ours, we may find an even lower percentage of Koos grade IV tumors. For example, in Iwai et al. [29], which is a large series with a long-term follow-up, the percentage of Koos grade IV tumors is 10.5%. Thus, we believe that it will be less and less uncommon to have a low percentage of Koos grade IV tumors in modern series of VS, especially in teams offering radiosurgery in addition to combined approaches.

### Facial nerve outcome in the management of large VS

For microsurgery of large VS, it has been well accepted that the size of the tumor is the main predictor for preservation of the facial nerve, both anatomically and functionally [31, 64]. The risk for facial nerve dysfunction in patients with VS larger than 3 cm is 6-fold greater than in patients with smaller tumors [73]. In Samii et al.’s series of large VS [64], even though a facial nerve function considered excellent/good was achieved in 75%, subgroup analysis shows that only 25% of patients retained H–B I facial nerve function after surgery. Following total microsurgical resection of large VS (larger than 3 cm in size), facial nerve function preservation (H–B I or II) was achieved in 27–58% [3, 31, 40, 62, 63, 79] in the major reported series.

A meta-analysis published by Gurgel et al. [21] analyzed facial nerve outcomes after surgery for large VS depending on the type of surgical excision. They found that H–B I or II were reported in 65.2% of the 601 retrosigmoid approaches included in their study. Furthermore, facial nerve outcomes were not significantly different using translabyrinthine or retrosigmoid approaches, but showed statistically better outcome, compared with the extended translabyrinthine approach. In the same meta-analysis [21], the authors found a strong and significant association between the degree of resection and outcome. Of the 80 patients with subtotal resection, 92.5% had good facial nerve outcomes, compared with 74.6% and 47.3% of those who received near-total resection and gross total resection respectively.

Subtotal resection achieves better preservation rates, between 82 and 88% (H–B I and II) [37, 50], and even almost 100% in some reports [27]. When using a combination of microsurgery and radiosurgery for large VS, authors reported facial nerve function preservation (H–B I and II) ranging between 85.7 to 95% [15, 27, 49, 50, 69]. For example, in the series by Van de Langenberg et al. [69], after planned subtotal resection, good facial nerve outcomes (H–B I or II) were reported in 94% of the cases after microsurgery and GKRS. In another series by Pan et al. [49], outcomes were reported after intracapsular decompression (group I) or radical extracapsular resection (group II). For group I, the pre- and post-surgical tumor volumes were 17.5 ± 1.1 cm^3^ and 9.35 ± 1.02 cm^3^ respectively, and for the group II they were 16.4 ± 0.95 cm^3^ and 1.1 ± 0.14 cm^3^ respectively. For group I, 90% of the patients retained excellent facial function (H–B I or II). For group II, only 35% retained excellent facial nerve function (data statistically significant).

It is worth noting that most of the series reporting facial nerve function after microsurgical resection, “excellent” or “good” results usually include patients in H–B I or II (or sometimes reaching H–B I to III), which from the functional point of view and quality of life of the patients is not similar to “normal” facial nerve function (i.e., H–B I).

For small- to medium-sized VS (Koos grades I to III) treated with first-line GKRS, Regis et al. [60] recently reported their experience in a very large cohort of 3,050 patients, with 2,336 having a minimum of 3 years’ follow-up, over a 20-year period of experience. Although the global rate of transient facial palsy was less than 0.5% in the entire series, there was a definite trend in improved facial nerve outcomes with experience and new technology. Since the introduction of GKRS robotization, facial nerve paresis has virtually disappeared in their series. This observation is in agreement with our experience in treating VS with GKRS, since the introduction and use of the Leksell Gamma Knife® Perfexion™ in Lausanne, with no permanent facial nerve impairment in our whole series.

Thus, our current results in patients with large VS, with 100% facial nerve functional preservation, H–B I outcome, and a high level of hearing preservation, compete favorably with the existing literature on the surgical management of large VS. This supports the assumption that our combined approach warrants strong consideration and further evaluation as a preferred option for those patients with larger tumors.

### Hearing outcome in the management of large VS

The size of VS does not necessarily correlate with the presence of serviceable hearing at the time of presentation [68]. Large tumors can also present with good hearing levels. Hearing preservation rates following microsurgical resection in large VS vary between 0 and 29% [12–14, 25, 63, 64, 73]. In one of the publications by Samii et al. [64], the probability of hearing preservation after total excision was estimated to be 11%. In a surgical series of 54 patients (75.9% total removal) with preserved hearing at the time of surgery and VS measuring ≥ 20 mm in extrameatal diameter (16 patients with ≥30 mm), hearing preservation was achieved in 53.7%, but only 31% had maintenance (or improvement) of hearing at the same level as before surgery [71]. In a recent systematic review on VS surgery, Ansari et al. [2] reported on 127 patients with tumors with a maximal diameter larger than 3 cm. This report did not provide the stratified data on the extent of tumor removal across the series. Nevertheless, hearing preservation was found to be possible in only 28.3% [2]. Di Maio et al. reported on a series of 46 large tumors (≥ 30 mm); 28 patients had hearing preservation surgery and of these patients, 6 (21.4%) had hearing preservation after surgery [10].

By comparison, for small to medium-sized tumors, radiosurgery data show a hearing preservation rate ranging between 38% and 94% [6, 23, 34, 36, 38, 48, 56, 70, 74]. A recent meta-analysis of published literature on GKRS for VS included 28 studies published between 2007 and 2011, with 3,233 patients [61]. The average prescription dose was 12.4 Gy. The preservation of serviceable hearing was on average 66.45%, with a mean follow-up of 51 months. The authors also made a comparison with similar studies [45, 72, 75–78], showing the preservation of serviceable hearing in 20–57% of the patients. In a systematic review, Yang et al. [77] included 45 articles, with 4,234 treated patients, and a median reported follow-up of 35 months. The mean GKRS dose was 14.2 ± 2.4 Gy. The overall hearing preservation rate was 51%, irrespective of radiation dose, tumor size or patient’s age. The authors concluded after a more detailed analysis that a marginal dose less than 13 Gy was associated with a higher rate of preserved hearing.

Recent literature reveals better hearing outcomes with the subtotal excision of large VS, with or without additional radiosurgery. Van de Langenberg et al. [69] reported hearing preservation in only one of 4 patients with serviceable presurgical hearing. In another series of 11 patients who underwent intracapsular decompression of VS followed by GKRS, Pan et al. [49] reported hearing to be preserved in all patients, even though surprisingly, they reported only 89% facial nerve preservation in the same series.

The results of our series are also in line with these reports, showing hearing preservation that is comparable with those results, or even better.

### Tumor control

Vestibular schwannomas are known to have a small annual rate of growth [65]. Despite this, recurrences may occur between 7 and 11% when surgical resection is considered to be total [40, 62], and between 7 and 53% in subtotal resection [19, 32, 33, 54]. There is a clear relationship between the residual tumor volume and further recurrence. This observation is further confirmed by a recent series by Vakilian et al. [67], in which the authors found that all VS with a postsurgical volume greater than 2.5 cm^3^ recurred.

Tumor control rates of GKRS in VS are reported to be as high as 97.5% of the cases, with a median decrease in size of 40% at 7 years’ follow-up [60]. A recent meta-analysis of published literature on GKRS for VS, which included 28 studies published between 2007 and 2011 and 3,233 patients reported an average tumor control of 92.7%, after a mean follow-up of 51.24 months [61]. The authors also made a comparison with similar studies [45, 72, 75–78], showing 81–100% tumor control for tumors ranging from 2.7 to 4 cm^3^.

The reported tumor control rate for combined approaches ranges between 79% and 100%, with our study reporting 91.6% (Table 3). In this sense, our local control is perfectly comparable with what has been published to date. Nevertheless, an analysis of actuarial tumor control rates would show lower rates (i.e., 90.7%, and actuarial rates of 94% at 1 year, 88% at 2 years, and 77% at 2.6 years, which further remained stable over time) than in large series of upfront GKRS for VS. However, at this stage of our analysis, the interpretation of the actuarial control rate for the current follow-up period, on a small sample size and a small number of failures (only 3 cases) should be cautiously interpreted. As there was no selection bias regarding the inclusion of patients treated with our combined approach, several other hypotheses may account for this finding. These large tumors could be biologically more aggressive, a fact that cannot yet be proven with standard neuropathological examinations. Although not performed systematically in our series, many pathological samples have been tested for Ki-67 or MIB1 indexes, and did not show any abnormal increase in cell proliferation with these markers (data not shown). Advances in pathological evaluation, searching for specific biological markers for the possible aggressive nature of VS, may help to better understand the specific situation of these failures. Another important factor could be the learning curve of the neurosurgical technique, from the point of view of the ability to have the thinnest and uniform residual capsule that is left in place covering the facial and cochlear nerves; the learning curve for the GKRS planning in its ability to achieve an optimal plan in these difficultly shaped target volumes could also be a factor of failure. Of note, the 3 cases of failures happened in patients treated early in this series (i.e., patients numbers 3, 9, and 11). Increased experience with subtotal excision and optimized planning radiosurgical strategies could reduce the incidence of treatment failures with this approach.

**Table 3:** Summary of the main series published in the literature

Reference	Number of patients	Follow-up (months)	Facial nerve preservation (%)	Cochlear nerve preservation (%)	Tumor control (%)
Iwai et al. [27]	14	32	85.7	NA	79
Park et al. [50]	8	68.8	NA	NA	100
Yang et al. [78]	61	53.7	95	NA	93.5
Fuentes et al. [15]	8	46	87.5	NA	100
Van de Langenberg et al. [69]	50	33.8	94	25 (1/4)	90
Pan et al. [49]	18	57	89	100 (11/11)	100
Iwai et al. [28]	40	66	95	42.9 (6/14)	90
Radwan et al. [53]	22	28	87	NA	100
Present study	32	29	100	76.9 (10/13)	91.6

### Combination of surgery and GKRS for large VS

Surgery for large VS is currently considered the gold standard. The number of patients presenting with large VS is decreasing, as imaging diagnosis and management are performed earlier in smaller tumors. For example, in our series, when all consecutive patients managed in our center were considered, only 25 patients out of 217 (11.5%) presented with VS too large for upfront GKRS, and underwent planned subtotal surgical excision as part of a combined approach. This low number reflects the incidence of large VS in a skull-base neurosurgical center rather than low recruitment, as almost 90% of our patients could be treated with upfront radiosurgery, and VS represent more than 25% of our GKRS activity.

Although it seems that planned partial resection followed by radiosurgery has become an increasingly popular approach in the neurosurgical community, very few centers have reported their results, as recently reviewed by Iwai et al. [28]. Pan et al. [49] compared two groups of patients with large VS, namely partial excision followed by GKRS (group 1 with 18 patients) versus total excision (group 2 with 17 patients). An excellent facial nerve outcome (H–B I or II) was achieved in 89% in group I and in 35% in group II; hearing preservation was 100% in group I and 0% in group 2. In Van de Langenberg’s series of 50 patients with the same surgical strategy [69], an excellent facial nerve outcome (H–B I or II) was achieved in 94% and hearing was preserved in 1 out of 4 patients. A recent systematic review by Brokinkel et al. [4] analyzed 6 studies of GKRS following subtotal resection. There were 159 patients with a tumor diameter more than 2 cm. After a mean follow-up of 15 months, good facial nerve function (H–B I or II) was achieved in 94%, whereas serviceable hearing preservation was achieved in 11.6%. Tumor growth control was achieved in 93.8%. Recently, Radwan et al. [53] have presented their results in a series of 22 patients treated with planned subtotal resection. Seventeen of them had radiosurgery and 5 wanted to undergo a “wait-and-scan” strategy for the remnant. Immediately after surgery, 68% of the patients had good and moderate facial nerve function; however, 32% had significant facial weakness (H–B IV or even higher). After a mean postoperative follow-up period of 28 months, 86.4% presented excellent facial nerve function, the former still including, however, H–B grades I and II. Regarding hearing preservation, 36% had serviceable hearing, including 6 patients with G–R 2 and 2 patients with G–R 1; one had decreased hearing function at 6 months after radiosurgery (passing from G–R 2 to G–R 4). Facial numbness was encountered in 18% immediately after surgery, showing improvement within the follow-up course; 9% experienced temporary dysphagia and dysarthria. The mean time between surgery and radiosurgery was 9.5 months. In this series, the radiosurgery management was heterogeneous, with 9 cases undergoing GKRS and 7 hypofractionated regimens [53].

Again, compared with those results, our series of 32 patients with large VS provides optimal results, with a normal facial nerve function (H–B I) outcome in 100% of the cases, and a high rate of hearing preservation (77.8% of the patients in G–R 1 preoperatively remained at G–R 1, and 92% of the patients with residual hearing G–R 1–3 preoperatively remained in G–R 1–3 postoperatively).

Achieving the optimal residual tumor volume for radiosurgery after microsurgical resection is not always easy, especially when cochlear nerve function preservation is the goal. This is exemplified in our series, where we found in 3 patients that the residual volume was too large for safe GKRS and we therefore decided on a second planned subtotal surgery. The facial nerve outcome was identical after the second surgery and GKRS (H–B I), but 1 patient lost hearing. However, the benefit of a staged resection needs to be balanced with the morbidity associated with a second hospitalization and craniotomy. If a single-stage excision can achieve the same outcome, this situation remains preferable [22]. In our experience, the 3 patients who needed staged surgery clearly reflect the learning curve of the technique that we have developed.

The inherent philosophy of combining surgery with radiosurgery needs a good understanding of both the therapeutic steps and its respective safety–efficacy evaluation. Although the microsurgeon can avoid direct dissection between the tumor capsule and the facial and cochlear nerves and improve the functional outcome of resection with a “nerve-centered” tumor surgery approach, the GKRS surgeon needs to appreciate that treatment planning may be more difficult because of the modification of local conditions as a result of surgery, to achieve a treatment plan that allows for the best functional outcome, as for smaller tumors. Both the extent of the planned microsurgical resection and the radiosurgical management, including its timing, are key to the success of this approach. There are 2 strategies for the subtotal resection of large VS. One consists in a planned subtotal resection in which the neurosurgeon focuses the technique on cranial nerve preservation and on resecting only the amount of tumor that is needed to render the residual tumor volume compatible with radiosurgery. This yields to the best functional outcome, as recently reviewed by Iwai et al. [28] and reported in our series. Another approach consists in performing a near-total extirpation, aiming to leave only a small tumor remnant, usually at the level of the internal acoustic porus, considering that it is the location in which the facial nerve is particularly at risk for injury and functional impairment. This approach, whose goal is also a better functional outcome, has proven to be less favorable. For example, in the recent publication by Jeltema et al. [30], aimed at near total extirpation of large VS with salvage radiosurgery only when the remnant is showing growth, normal facial nerve function (H–B I) was achieved in only 57.7% of the cases, whereas 32.7% of the patients had mild (H–B II–III) facial function impairment after surgery, and 9.6% had severe (H–B IV–V) impairment; there was no mentioning of the hearing status. Thus, this later approach does not represent a “real” planned subtotal extirpation, and functional results might have been better if a larger residual tumor had been left in place. The subsequent, and related, issue concerns the timing of radiosurgery after subtotal resection of VS. Although Jeltema et al. [30] are in favor of salvage radiosurgery only when the remnant is growing, arguing that it was needed in only 13% of their series, we and others [28] believe that in planned subtotal resection, when a larger piece of VS is left in place, GKRS should take place in the months following surgery, as part of a combined approach. We consider that the residual tumor is at a high risk for further regrowth and we prefer to perform GKRS when the residual VS has a volume and anatomical relationship that is suitable for optimal radiosurgery dosimetry planning and treatment.

### Study limitations

There are several limitations of the present study. The first is the short follow-up period following surgery and GKRS; there is a need for further evaluation and re-confirmation of the current clinical and neuroimaging results presented in this study. Second, owing to the small sample size, the statistical power is limited. Third, there was a relatively short interval between the surgical resection and GKRS targeting, which may not have offered enough time for good visualization of the tumor and thus allow optimal GKRS targeting. Fourth, there is an ongoing debate concerning the regrowth of the residual tumor after surgery and the possible need for further irradiation. In our center, the current strategy is to offer a combined treatment to the patient from the very beginning, including both a planned subtotal surgery and further GKRS on the residual tumor several months later.

## Conclusion

The results of this series of large VS show that planned subtotal resection followed by GKRS has an excellent clinical outcome with regard to facial nerve (100% H–B I) and cochlear nerve (77.8% G–R 1; 92% G–R 1–3) function preservation. Surgery for large VS has significantly changed from the total excisions performed previously to a “nerve-centered” tumor surgery approach. This shift in treatment paradigm will need to be confirmed with long-term results following the GKRS. As these long-term results emerge, this method of combining subtotal surgery with GKRS in a planned manner warrants strong consideration as the preferred option for patients with large VS. Indeed, our results with planned subtotal resection followed by GKRS in large VS compare favorably with the results obtained with first-line GKRS in small- and medium-sized VS, allowing to match similar expectations for patients with larger VS needing surgical removal.
